# Implementation science infrastructure in U.S. academic institutions: a convergent mixed-methods study

**DOI:** 10.1186/s43058-026-01046-2

**Published:** 2026-07-16

**Authors:** Carolina Mejia, Megan M. Oakes, Zoe E. Waddell, Sahar Shibeika, India Rockett, Stephanie S. Sperry, John A. Fuller, Lesley A. Skalla, Connor Drake, Devon Check, Heather A. King, Leah L. Zullig

**Affiliations:** 1 Department of Population Health Sciences, Duke University School of Medicine, 411 West Chapel Hill Street, Suite 600, Durham, NC 27701 USA; 2 Department of Health Policy and Management, University of North Carolina, Chapel Hill, NC USA; 3 Nell Hodgson Woodruff School of Nursing, Emory University, Atlanta, GA USA; 4 Duke University Medical Center Library & Archives, Duke University School of Medicine, Durham, NC USA; 5 Center of Innovation to Accelerate Discovery and Practice Transformation, Durham VA Health Care System, Durham, NC USA; 6 University of Michigan Medical School, Ann Arbor, MI USA; 7 Division of General Internal Medicine, Duke University School of Medicine, Durham, NC USA

**Keywords:** Implementation science, Academic health centers, Research infrastructure, Sustainability, Capacity building, Workforce development, Leadership succession, Mixed-methods, Metrics

## Abstract

**Background:**

Interest in implementation science (IS) has grown rapidly in recent years, leading to the development of academic centers and programs across the United States (U.S.). Limited information exists regarding how these entities are structured, funded, and sustained. This study characterizes the infrastructure, funding models, service offerings, and organizational structures and practices of IS centers and programs within U.S. academic institutions.

**Methods:**

We conducted a convergent mixed methods study using a structured web-based review and a REDCap survey. Targeted Google searches of .edu and .org domains were used to identify IS centers and programs based at U.S. academic medical and public health institutions. Forty-eight centers met the inclusion criteria after screening. Website data included institutional affiliation, leadership structure, focus areas, partnerships and service offerings. Program leaders from identified centers were invited to complete a survey examining funding sources, success metrics, workforce development, partnerships, and leadership succession. Quantitative data were analyzed descriptively, and open-ended responses were analyzed using rapid qualitative methods. Findings were integrated during interpretation.

**Results:**

The 48 centers and programs were distributed across all major U.S. regions, with 54.2% located at public institutions. Research combined with mentorship and/or teaching was the most common focus area (77.0%). Training (54.2%) and consultation (45.8%) were the most reported service offerings, whereas only 27.1% publicly described community engagement support. Among 12 of 13 survey respondents with complete funding data, grants were the most frequently reported funding source (83.3%), followed by institutional support (50.0%), with limited funding diversification. Integration of quantitative and qualitative findings suggested that workforce development, mentorship, and partnerships were viewed as important strengths among survey participants, while sustainability challenges were linked to reliance on external funding, staffing constraints, and limited succession planning. Quantitative findings related to funding concentration and traditional success metrics were reinforced by qualitative themes describing Clinical and Translational Science Award (CTSA) dependence, workforce challenges, and misalignment between academic incentives and long-term implementation impact.

**Conclusions:**

Academic IS centers and programs play an important role in workforce development, mentorship, training, and implementation support but continue to rely substantially on external funding to support core activities. Findings highlight the need for diversified funding strategies, stronger institutional investments, proactive succession planning, and broader impact-oriented success metrics that better capture long-term implementation impact and systems change.

**Supplementary Information:**

The online version contains supplementary material available at https://doi.org/10.1186/s43058-026-01046-2.

Contributions to the literature

- To our knowledge, this study provides one of the first national snapshots of how academic implementation science (IS) centers and programs are structured, funded, and evaluated across the U.S., addressing an important gap in the literature on IS infrastructure.
- Findings highlight the central role of training, mentorship, and consultation services within IS infrastructure while identifying sustainability challenges related to reliance on external grant funding and concentrated funding streams.
- Results offer practical insights into workforce development, funding diversification, mentoring practices, leadership succession, and partnership models while underscoring the need for broader impact-oriented success metrics that are better aligned with the applied values of IS.

## Background

Implementation science (IS), as a discipline, seeks to systematically close the gap between what we know should be done to improve population health and what is done in practice. The application of evidence-based programs, policies, and practices in healthcare and community settings often lags behind the generation of research findings, limiting the potential population health impact of scientific discoveries [1]. It has been reported that a minority of research findings are ever translated into real-world settings, and that when they are, it takes an average of 17 years for new research findings to be adopted into routine practice [2]. By the time these interventions reach the “real-world”, they may already be outdated or less effective due to changes in healthcare systems, population needs, or advancements in medical knowledge. This is often referred to as the “know-do gap” [3]. Closing this know-do gap is crucial for realizing the full potential of evidence-based programs, policies, and practices in real-world contexts. Doing this effectively requires infrastructure to support implementation and IS research to translate research into practice.

One important strategy for closing the know-do gap is strengthening IS infrastructure. In this study, IS infrastructure refers to the organizational structures, resources, expertise, and support systems that enable the conduct, training, dissemination, and application of IS research and practice. Within academic medicine, this IS infrastructure commonly occurs in the form of centers, programs, cores, institutes, research collaboratives, and cross-institutional partnerships. Although these organizational forms vary in scope and function, they serve as hubs for training, research, mentorship, consultation, interest-holder engagement, pilot funding, and the dissemination of implementation methods and evidence-based practices [4]. Such infrastructure is essential in academic medicine because it helps identify and address barriers that hinder the uptake of evidence-based programs, practices, and policies [5].

In recent years, funding opportunities and institutional investments focused on IS infrastructure have expanded substantially. National dissemination and implementation (D&I) capacity-building initiatives, including Clinical and Translational Science Award (CTSA)-supported training programs, Patient-Centered Outcomes Research Institute (PCORI)-funded D&I training initiatives, the Training Institute for Dissemination and Implementation Research in Health (TIDIRH), and implementation science fellowships, have further contributed to expanding the IS workforce and research infrastructure [4, 6, 7]. Universities across the U.S., including the University of Washington, the University of North Carolina, and Washington University in St. Louis, have developed specialized IS centers and training programs to build capacity among researchers and practitioners [8, 9]. Across funding agencies and disciplines, there has been a notable increase in interest in embedding IS into academic medicine, as demonstrated by the rise in dedicated funding mechanisms, educational programs, and cross-institutional collaborations [10].

While IS infrastructure is also being developed within health systems, state agencies, the pharmaceutical sector, often to support applied implementation, academic centers play a distinct and foundational role in advancing the science itself. These centers contribute to methodological innovation, theory development, and the generation of rigorous evidence that extends across health and community settings. They also serve as primary training grounds for the next generation of implementation scientists, positioning them as central to long-term development and evolution of the field [5, 10].

Despite the growing interest in and need for IS infrastructure, there remains limited empirical literature describing how IS centers and programs are organized, funded, sustained, and operationalized. One potential reason for this gap is that detailed information about IS infrastructure is not routinely or systematically reported in scholarly literature and is often available primarily through institutional websites and other gray literature sources.

To address this gap, we used a structured web-based approach to review websites of U.S.-based IS centers and programs. Our goal was to characterize programs’ organizational structures and funding models, along with their missions and areas of focus. By synthesizing this information, we provide insights that can inform how IS centers and programs are structured and sustained and offer practical considerations for strengthening IS capacity and supporting the effective implementation of evidence-based interventions to improve population health outcomes.

## Methods

### Study design

We conducted a convergent mixed-methods study to characterize the structure, funding, and operational functions of IS centers and programs in the U.S. In a convergent mixed-methods design, quantitative and qualitative data are analyzed separately and then integrated during interpretation to provide a more comprehensive understanding of the phenomenon under study [11, 12]. We integrated data by comparing quantitative findings from the web-based review and survey with qualitative themes derived from the open-ended survey responses to identify areas of convergence, complementarity, and divergence that informed the overall interpretation of the findings. Specifically, quantitative findings from the web-based review and REDCap survey were analyzed using descriptive statistics, while open-ended survey responses were analyzed using rapid qualitative analysis. The quantitative and qualitative findings were then integrated through side-by-side comparison and interpretation to identify areas of convergence and complementarity and to develop a comprehensive understanding of implementation science infrastructure, funding, sustainability, workforce development, and organizational practices. Additional details regarding the study design and integration procedures are provided in Supplementary Figure S1.

The study integrated data from a structured web-based review conducted in 2022 and a REDCap survey administered between 2024 and 2025. The web-based review provided quantitative data describing organizational characteristics, partnerships, and publicly described service offerings and established the initial sampling frame. The survey generated both quantitative and qualitative data, including information on funding sources, measures of program success, workforce development, leadership transitions, and sustainability. Quantitative and qualitative data were analyzed independently and subsequently compared and integrated during interpretation using a side-by-side approach. Qualitative findings were used to contextualize and explain quantitative patterns related to funding, sustainability, organizational structure, and program operations. Although there was a temporal gap between data sources, the survey allowed respondents to verify, update, and supplement information obtained through the web-based review and provide a more current perspective on organizational practices and sustainability.

### Identification of IS centers and programs (web-based review)

In collaboration with a medical center librarian (LAS) with experience in grey literature searching we developed and refined a structured Google-based web search strategy to identify websites of existing academic IS centers and programs. The librarian contributed methodological expertise in search strategy development and advised on procedures for website identification, screening, and documentation to enhance the transparency and rigor of the search process.

### Eligibility criteria

Centers and programs were deemed eligible for inclusion if they: (1) were affiliated with an academic institution (e.g., school of medicine, school of public health, or university-based college, department, or institute); (2) had a focus on one or more aspects of implementation science (implementation, dissemination, or translational research); and (3) functioned as an organized or shared resource (e.g., hub, collaborative, core, group, unit, etc.). Entities identified during screening were excluded if they primarily focused on quality improvement or performance improvement initiatives without an implementation science component; or structured as academic departments or schools rather than organized centers or programs. Eligibility determinations were based on publicly available website content using predefined criteria. In cases of uncertainty, classifications were resolved through team discussion.

Because the boundary between IS and quality improvement is not always clearly defined in practice, borderline cases were reviewed carefully during screening. Our team erred on the side on inclusion. Programs were classified as implementation science-focused when website content explicitly referenced implementation, dissemination, translational, or delivery science research activities, frameworks, or methodologies. Programs focused primarily on operational quality improvement or institutional performance initiatives without a stated research or dissemination focus were excluded.

### Search strategy and screening

The medical center librarian, in consultation with the author team, developed the Google search strategies. We conducted six unique targeted searches, limited to Uniform Resource Locators (URLs) ending with “.edu” or “.org,” for a total of 12 searches. Search strings included various terms related to implementation, dissemination, outcomes, translational, or delivery science/research combined with Boolean AND to terms for centers and programs (e.g., universities, colleges, centers, departments, programs, collaboratives, hubs, or cores). The complete search strategy is provided in Supplementary Table S2 and is also available through an open-access repository (http://hdl.handle.net/2193/5X21TG037).

The searches were conducted between November 11–22, 2022. The objective was to identify publicly visible IS centers and programs rather than conduct a systematic review; therefore, screening was limited to the first 20 entries per search string, yielding 240 records. Because Google search results may vary over time, by user, and by search environment, the search strategy was designed to prioritize transparency and practical discoverability over strict reproducibility typically associated with systematic reviews.

**Fig. 1:**
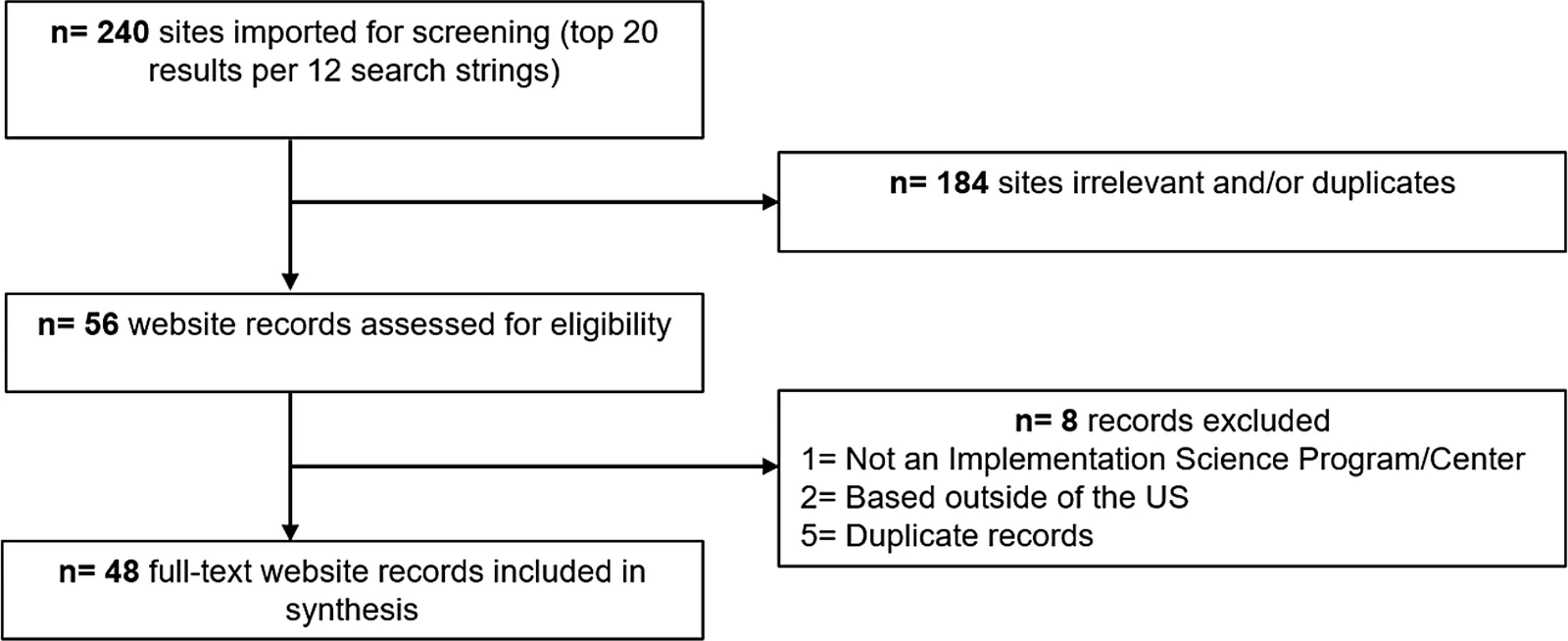
Flow diagram of web-based identification and selection of implementation science centers and programs

To reduce duplication across searches, potentially relevant websites identified through Google searches were tracked using a structured bookmarking approach adapted from published grey literature methods [13]. Bookmarks were organized by search strategy, allowing reviewers to identify previously screened websites, minimize duplicate review across search teams, and maintain a documented record of the search process. Google search settings were configured to display up to 100 results per page to facilitate screening efficiency, although only the first 20 results for each search string were reviewed. Search results were screened independently by two reviewers (SS, IR) with disagreements resolved by a third reviewer (LLZ). Of the 240 records, 184 were excluded during the initial screening. Fifty-six websites underwent full review, resulting in a final sample of 48 IS centers or programs eligible for data extraction (Fig. 1). The complete list of IS centers and programs included in the web-based review is provided in Supplementary Table S1.

### Data extraction

Data were extracted and organized using Microsoft Excel. The complete data dictionary, variable definitions, and coding framework used for data extraction and analysis are provided in Supplementary Table S3. Website details extracted included university name, program or center name, geographic location, website URL, funding source (if reported), and leadership point of contact. Data extraction was completed by (ZW, IR, JF, SS) and subsequently reviewed by LLZ for completeness and consistency.

### Operational coding and service offerings analysis

We characterized the primary focus area of IS centers and programs using publicly available website content. Focus areas included research (conducting or supporting IS research), teaching (formal coursework, curricula, workshops, or educational programming), and mentorship (trainees, fellows, or junior faculty). Each center was coded dichotomously (present/absent) for each focus area based on information provided on the website and linked pages when available. Centers could be assigned to multiple categories. When classification was unclear, coding decisions were reviewed and resolved through team consensus.

Service offerings were identified through review of website descriptions collected during the web-based review. Categories were defined a priori and included consultation, training and education, technical assistance, pilot funding, community engagement, and networking or collaboration activities. Each center was coded dichotomously (present/absent) for each service category based on information available on the website and linked pages when applicable.

Data were collected using standardized extraction forms and predefined coding definitions. Team members were trained on coding procedures before data extraction began, and entries were reviewed for completeness and consistency after initial coding. When possible, information was verified across multiple pages within institutional websites.

### REDCap survey

Between October 2024 and January 2025, leaders from 48 identified centers and programs were invited to complete an online survey using REDCap, a secure, web-based software platform [14]. Invitations were sent by personalized emails and included pre-populated information about each center and program based on our prior web-based review. Respondents were asked to verify, correct, and supplement this information and answer several brief open-ended questions regarding program sustainability, and perceived drivers of success. The survey required approximately 10–15 min to complete. Participation was voluntary, and responses were reported in aggregate to maintain confidentiality. This study was approved by the Duke University Health System Institutional Review Board (IRB #Pro00116595).

### Quantitative analysis

Quantitative survey data collected via REDCap were exported into Microsoft Excel and Python (version 3.13.3) for cleaning and analysis. Initial data validation included frequency checks to identify missing or inconsistent responses. Descriptive statistics were then generated using Python’s Pandas (version 2.2.3) and NumPy (version 2.3.5) libraries to summarize program characteristics, including funding mechanisms, organizational structure, leadership roles, and educational activities. Key program characteristics included institutional affiliation, public or private university status, research, teaching, and mentorship foci, and whether the program was identified as patient- or community-focused. Funding sources were categorized into primary and additional funding sources. Nine funding types were analyzed, including grants, institutional support, government contracts, tuition or course fees, endowments, philanthropic donations, industry partnerships, consulting services, and “other”. Similarly, we analyzed measures used to track program success and the performance indicators respondents ranked among their top three program priorities. Summary statistics were stratified by institutional type (public vs. private) and visualized using bar charts to illustrate the distribution of primary and secondary funding sources and success measures.

### Qualitative analysis

Open-ended responses to three survey questions were analyzed using a structured matrix approach informed by Rapid Qualitative Analysis (RQA) methods [15]. The questions explored (1) staff recruitment, retention, and development; (2) leadership transition planning; and (3) additional insights, challenges, or successes. Responses were brief (1–5 sentences on average) and well-suited to RQA, emphasizing efficiency and key concepts while preserving analytic rigor in applied health services research. Data were organized into summary matrices by questions and respondent ID. One team member (CM) conducted the initial coding to identify key themes and subthemes, which were then reviewed and refined in discussion with two other team members (LLZ and MO). Themes were identified inductively based on patterns across responses. Consistent with a convergent mixed-methods design, qualitative findings were analyzed separately from quantitative data and integrated during interpretation through comparisons across data sources. The process allowed qualitative responses to contextualize results and provide additional information into sustainability, workforce development, leadership, and organizational structure [16]. The qualitative analytic framework, including domains, themes, and definitions, is provided in Supplementary Table S5.

## Results

### Characteristics of IS center and programs identified through web-based review

An overview of the characteristics of the 48 IS academic centers and programs resulting from the web review is presented in Table 1. Over half of the centers and programs were based in public institutions (54.2%). Research was included as a focus area in nearly all centers and programs and was often combined with mentorship and/or teaching. Fewer than half publicly described partnerships with healthcare systems or community groups.

**Table 1:** Characteristics of academic IS centers and programs identified through the web-based review (*N* = 48)

Characteristic	*N* (%)
**Institution Type**	
Public	26 (54.2)
Private	22 (45.8)
**Areas of Focus** ^**a**^	
Research + Mentorship	22 (45.8)
Research + Mentorship + Teaching	15 (31.2)
Research only	10 (20.8)
Teaching + Mentorship	1 (2.1)
**Partnered with a healthcare system**	
No	29 (60.4)
Yes	19 (39.6)
**Partnered with a community group**	
No	27 (56.2)
Yes	21 (43.8)

^a^ Focus area: Research = conducting or supporting implementation science research; Mentorship = formal mentoring of trainees, fellows, or junior faculty; Teaching = formal coursework, curricula, workshops, or educational programming

When stratified by institution type (not shown above), the most reported focus area across all centers was “Research + Mentorship” (45.8%), more prevalent among public institutions (53.8%) than private (36.4%). “Research + Teaching + Mentorship” was also frequently reported (31.2%), with similar proportions across public (26.9%) and private (36.4%) institutions. Approximately 20.8% of centers reported “Research Only” as their sole focus area, with a higher proportion among private institutions (27.3%) compared to public (15.4%). Only one center reported a focus on “Teaching + Mentorship” without research. Regarding external partnerships, private institutions reported more frequent collaboration with healthcare systems (45.5%) compared to public institutions (34.6%), whereas community group partnerships were similarly prevalent across both sectors (46.2% for public and 40.9% for private centers).

Publicly described service offerings.

Table 2 summarizes the service offerings publicly described by IS centers and programs identified through the web-based review. Training (54.2%) and consultation services (45.8%) were the most reported activities, whereas networking opportunities (12.5%) and pilot funding (4.2%) were reported less frequently. Overall, these findings suggest that workforce development, training, and implementation support represent core functions of IS centers and programs, whereas direct funding plays a limited role. Although implementation science emphasizes collaboration and community engagement, only 27.1% of centers publicly described community engagement activities on their websites.

**Table 2:** Publicly described service offerings among academic implementation science centers and programs identified through web-based review (*N* = 48)

Service Type	*N* (%)
Training	26 (54.2)
Consultation Services	22 (45.8)
Community Engagement Support	13 (27.1)
Technical Assistance	12 (25.0)
Networking Opportunities	6 (12.5)
Pilot Funding	2 (4.2)

Note: Centers and programs could be coded in more than one category; therefore, percentages do not sum to 100%. Absence of a service description on a website was not interpreted as absence of the service itself. ^a^ Service type was coded based on explicit descriptions available on publicly accessible center or program websites

Geographic distribution of centers and programs identified through web-based review (*N* = 48).

**Fig. 2:**
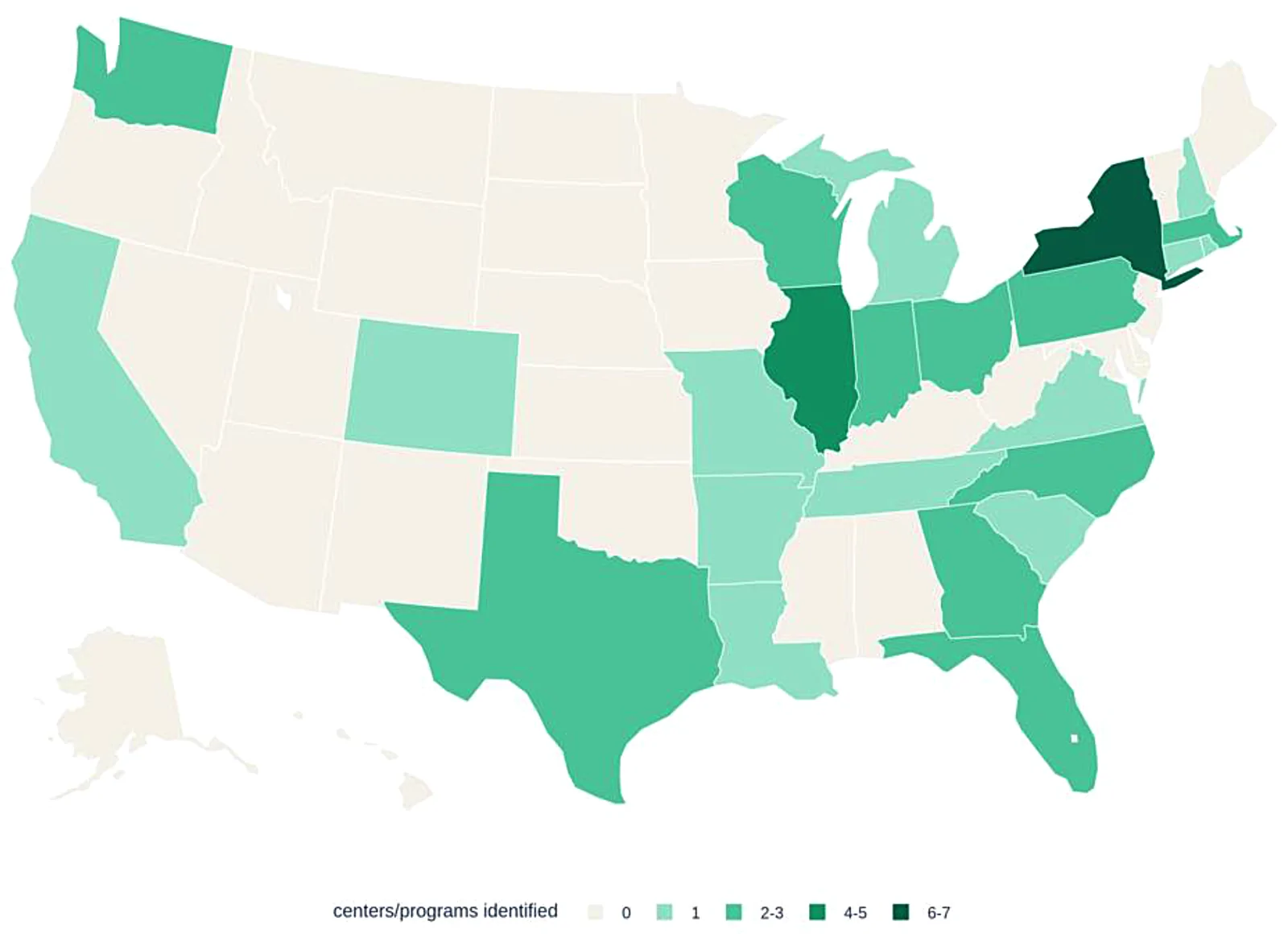
Geographic distribution of academic implementation science centers and programs identified through the web-based review (*N* = 48)

Figure 2 illustrates the geographic distribution of the 48 IS academic centers and programs included in this analysis. Centers and programs were identified across all major U.S. regions, with the Northeast accounting for the largest proportion (31.2%), followed by the Midwest (29.2%) and South (29.2%), while the West represented 10.4% of the sample.

### Funding sources and sustainability

In the REDCap survey, program leaders were asked to identify their center or program’s primary and secondary sources of funding (Table 3). Among the 13 survey respondents, one provided incomplete funding information; therefore, analyses of funding sources were based on 12 respondents. Among respondents, grants were the most frequently reported primary funding source (83.3%), followed by institutional support (50%). Funding by CTSA and Clinical and Translational Science Institute (CTSI) was publicly described among 12 of the 48 IS centers identified through the web-based review. Other sources, such as government contracts and tuition or course fees, were reported by only one center each, and no respondents selected endowments, philanthropic donations, or industry partnerships as primary sources of support. A small number of centers reported secondary or other sources of funding, including endowments, philanthropic contributions, and consulting services. These results highlight a relatively narrow and potentially vulnerable financial base for IS centers and programs, with a strong reliance on grants and institutional support.

**Table 3:** Funding sources among surveyed IS centers and programs (*n* = 12) ^a^

Funding Source	Reported as Primary Funding Source *n* (%)	Reported as Additional Funding Source *n* (%)
Grants	10 (83.3)	0 (0)
Institutional Support	6 (50.0)	4 (33.3)
Government Contracts	1 (8.3)	0 (0)
Tuition or Course Fees	1 (8.3)	0 (0)
Endowments	0 (0)	2 (16.7)
Philanthropic Donations	0 (0)	2 (16.7)
Contracts and Consulting Services	0 (0)	2 (16.7)
Industry Partnerships	0 (0)	0 (0)

Note: ^a^ Percentages are calculated using the number of respondents with complete data (*n* = 12). ^b^ Respondents could select more than one funding source category; therefore, percentages do not total 100%

When stratified by institution type, grants remained the most frequently reported primary funding source among both public (85.7%) and private (80.0%) institutions. Public institutions, however, described somewhat more diversified secondary funding streams, including endowments, philanthropy, and consulting services. Quantitative findings demonstrating heavy reliance on grants and institutional support were reinforced by qualitative responses describing concerns related to funding continuity, staffing limitations, and dependence on CTSA-related infrastructure.

### Measuring programmatic success

Table 4 summarizes responses to two related survey items designed to assess how IS centers and programs define and track program success. The first item asked respondents to select all performance measures used by their program, whereas the second asked respondents to rank, from among the measures they had selected in the first question, the three they considered most important to program success. Twelve of 13 respondents provided complete data for these items.

Respondents selected a range of measures for tracking success, with “project-specific grants” and “publications” used most frequently as primary measures (100% each), followed by “funding for mentorship or training opportunities” (83.3%) and “partnerships” (75.0%). These domains also appeared prominently as additional measures, with “project grants” most selected (41.7%). Less frequently reported indicators included “community collaborations” and “student or faculty engagement.” Three centers cited “other” primary measures of success, including “number and value of contracts”, “collaborations with internal research departments”, and “bibliometric” analyses. No centers reported “alumni impact” as either a primary or secondary success metric.

These quantitative patterns were further contextualized by qualitative responses emphasizing workforce development, collaboration, institutional visibility, and relationship-building as important indicators of programmatic success. Together, the findings suggest potential tension between conventional academic productivity metrics and the broader applied goals of implementation science infrastructure.

**Table 4:** Measures used to track and prioritize program success among surveyed IS centers and programs (*n* = 12) ^a^

Measure of Success	Used to Track Program Success *n* (%)	Ranked among Top 3 Priorities ^a^*n* (%)
Project grants	12 (100.0)	11 (91.7)
Publications	12 (100.0)	7 (58.3)
Funding mentorship/training opportunities	10 (83.3)	6 (50.0)
Partnerships	9 (75.0)	4 (33.3)
Center or program grants	7 (53.8)	3 (25.0)
Student/faculty engagement	6 (50.0)	2 (16.7)
Ongoing community collaborations	6 (50.0)	2 (16.7)
Other ^b^	3 (25.0)	0 (0)
Alumni impact	0 (0)	0 (0)

Note: ^a^ Percentages are calculated using the number of respondents with complete data (*n* = 12)

^b^ Respondents first selected all applicable performance measures used by their program and then ranked up to three measures as most important to program success. Percentages do not total 100% because respondents could select multiple categories. ^c^ Frequently ranked among top priorities reflects measures selected by respondents as one of their top three indicators of program success. ^d^ Other responses include: “number and dollar value of contracts”, “collaborations with other research-focused entities in our organization”, and “bibliometrics.”

**Fig. 3:**
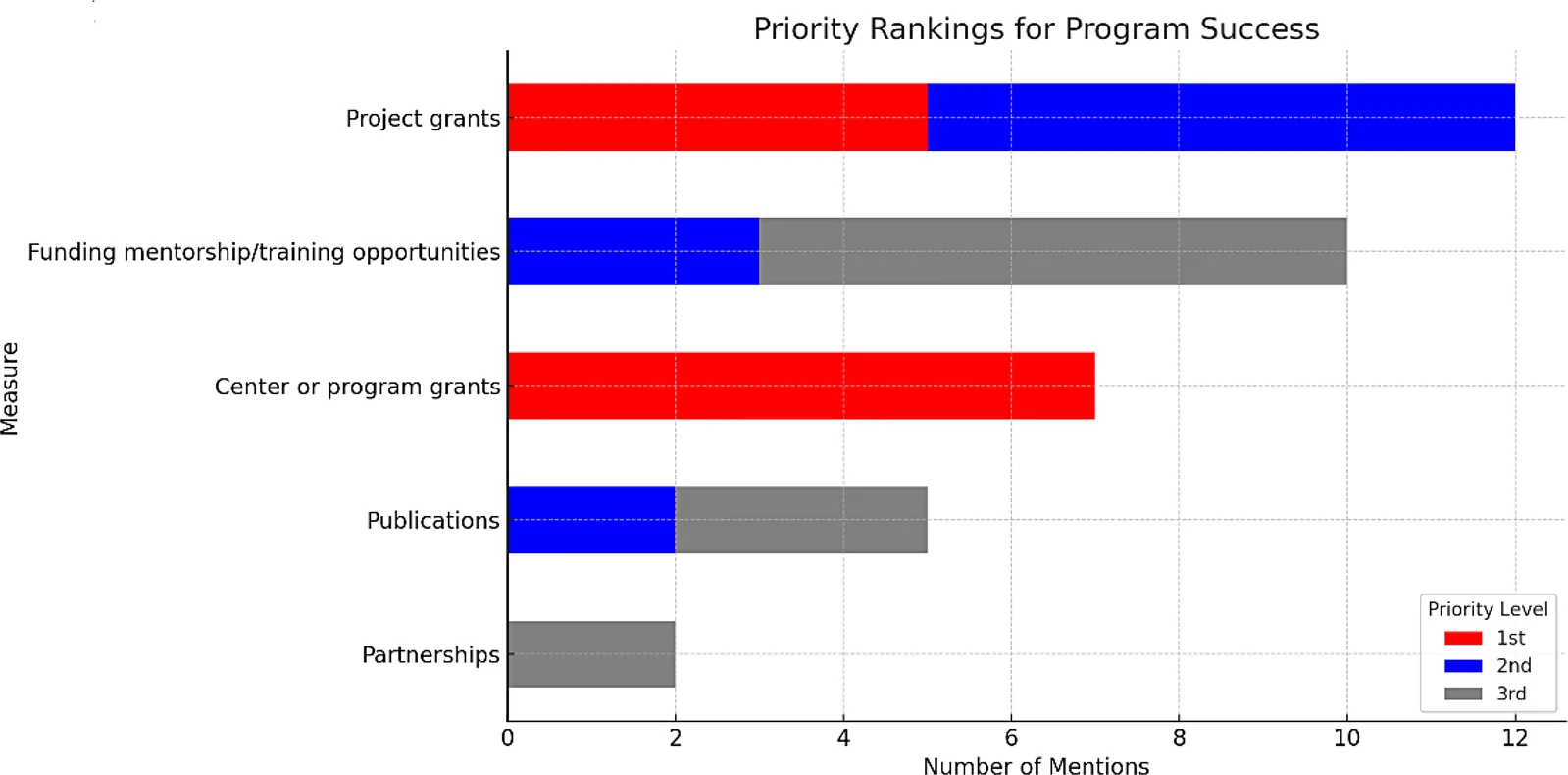
Priority rankings for program success based on survey responses (*n* = 12)

**Fig. 4:**
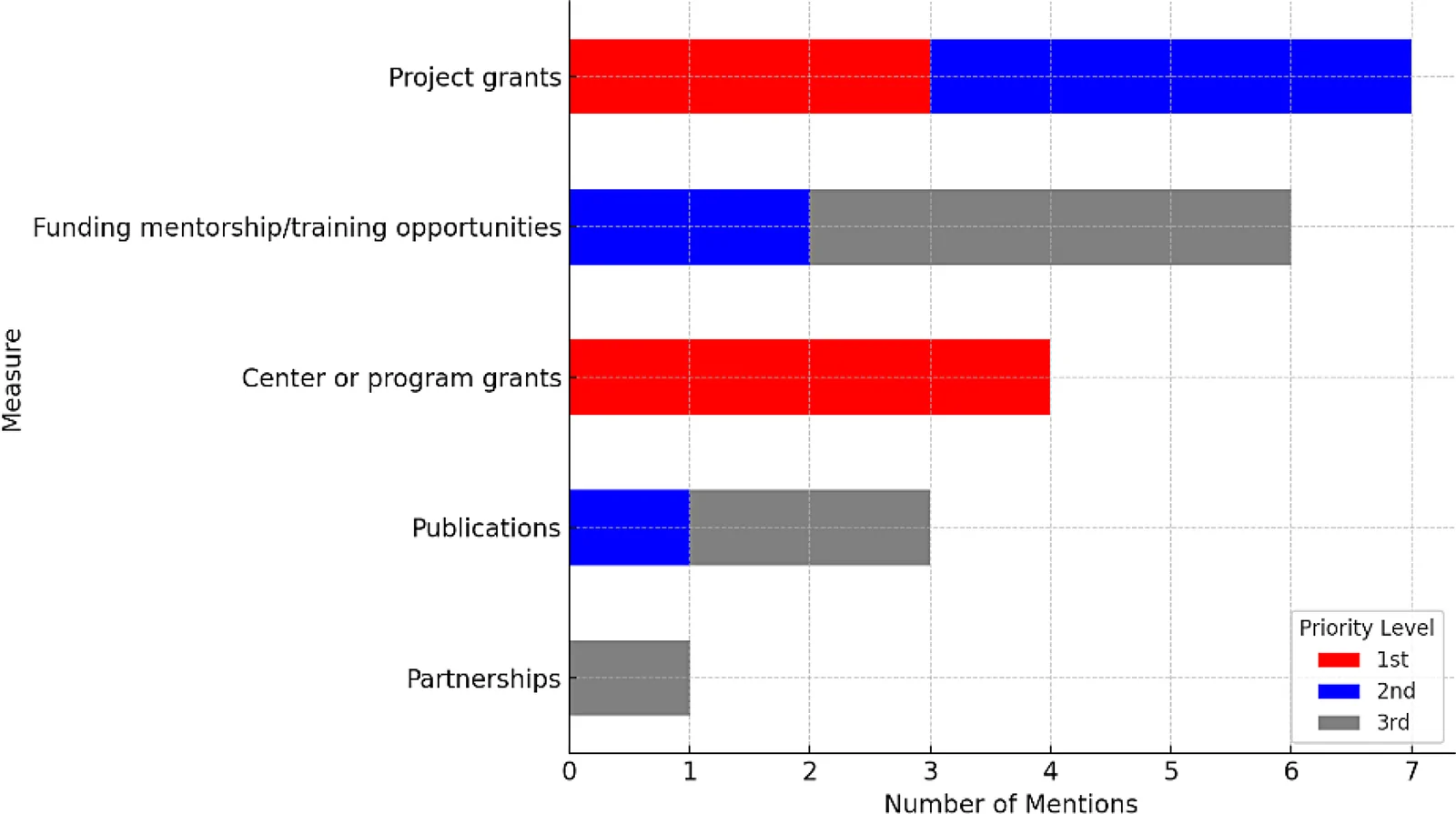
Priority rankings for program success for public institutions based on survey responses (*n* = 7)

**Fig. 5:**
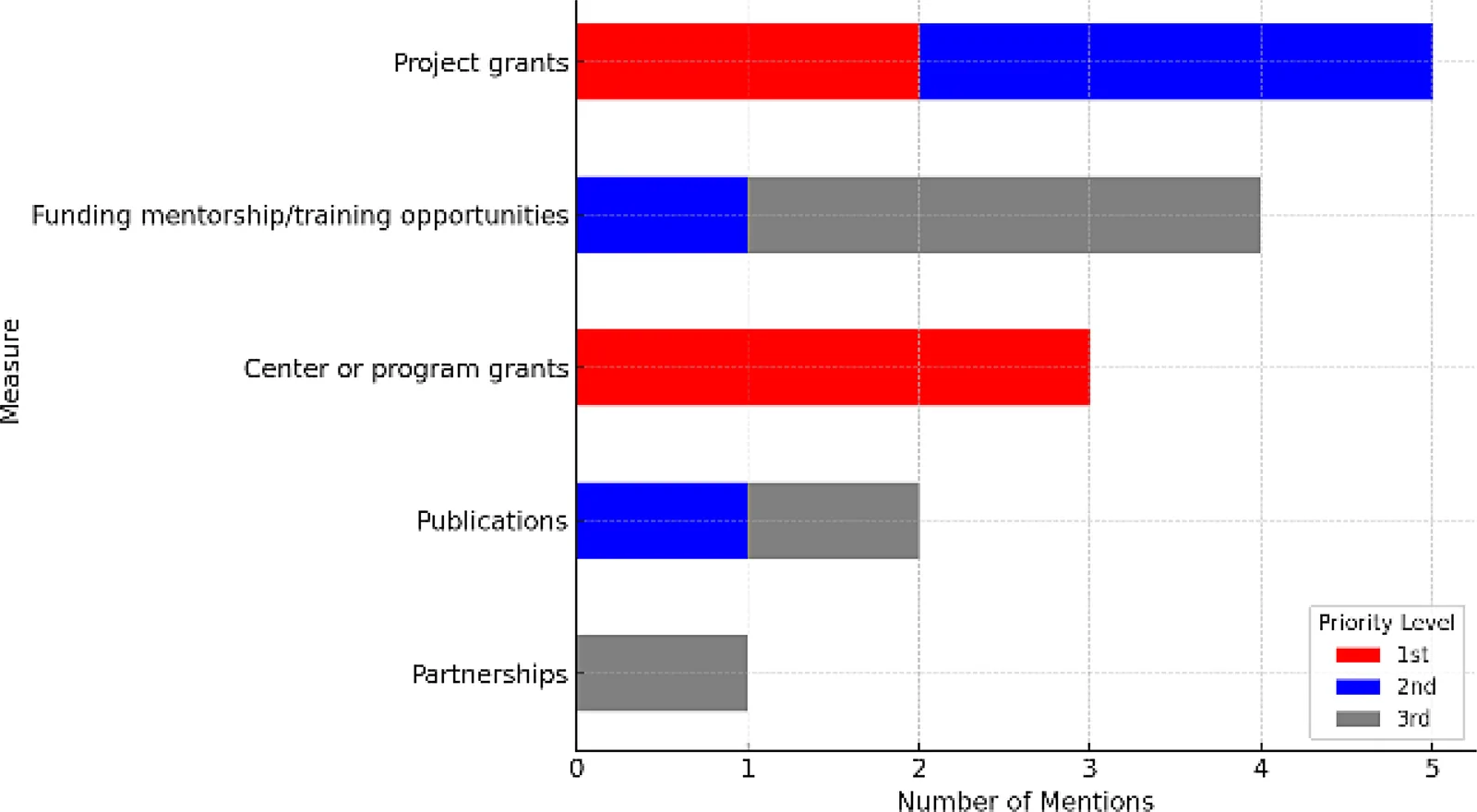
Priority rankings for program success for private institutions based on survey responses (*n* = 5)

Figures 3, 4 and 5 display the ranking data overall (Fig. 3) and by institutional type (Figs. 4 and 5). While traditional academic metrics dominated across both public and private institutions, the ranking exercise provided additional insights into which metrics are prioritized.

**Table 5:** Strategies for staff recruitment, retention, and development among IS programs and centers

Theme	Subtheme	Exemplary Quotes
Recruitment	Institutional structure (*n* = 2)	“Program recruitment, retention and development activities are coordinated with the Division of Implementation Science in the College of Medicine.” (ID 9) “We have a fairly robust staff professional development program, we survey our staff with pulse surveys to meet needs…” (ID 23)
Recruitment	Strategies (*n* = 3)	“Looking for best folks with aligned interests.” (ID 3) “Coordinate with university staff to get position descriptions together, jobs posted, candidates interviewed…” (ID 10) “Recruitment: 1) At conferences 2) Recruitment fairs 3) Through existing network…”(ID 17)
Retention	Retention via job structure (*n* = 2)	“We try to adjust the job titles as we are able, and to follow available promotion schedules as they exist…” (ID 10) “We have systems and structures in place to monitor on a monthly basis staff effort/grids to anticipate any funding cliffs… (ID 48)”
Professional Development	Training support (*n* = 2)	“Career development for these 3 people is funded through external grants based on availability of funds”. (ID 2) “We have trained several members of our team through our implementation science postdoctoral fellowship program.” (ID 7)
Professional Development	Supportive culture (*n* = 3)	“We focus on building a supportive culture that considers the whole person…” (ID 7) “Faculty are very supportive of finding and retaining (as well as can) good staff.” (ID 10) “We hold monthly action group meetings… a highly participatory approach that allows staff to partner, guide, and lead…” (ID 48)

### Survey participation and nonresponse assessment

Thirteen of the 48 identified implementation science centers and programs completed the REDCap survey (27.1%). One respondent provided only partial survey data; therefore, analyses of funding sources and measures of program success were based on 12 respondents. To assess potential nonresponse bias, we compared responding (*n* = 13) and nonresponding centers (*n* = 35) using organizational characteristics obtained from the web-based review. Responding and nonresponding centers were generally similar with respect to institution type, focus areas, and partnerships with healthcare systems and community organizations. Responding centers were somewhat more likely to publicly describe training activities (76.9% vs. 45.7%), consultation services (69.2% vs. 37.1%), and community engagement support (46.2% vs. 20.0%) than nonresponding centers (Supplementary Table S4).

### Qualitative findings

We analyzed open-ended responses from leaders of 13 IS centers to understand their strategies for building and sustaining IS infrastructure. We summarize responses to three key questions below.

#### Question 1: How does your program recruit, retain, and develop staff to support long-term success?

Respondents described several strategies for recruiting, retaining, and developing staff emphasizing workforce development as a central function of their centers. Common themes included professional growth, internal alignment, hiring practices, workplace culture, and long-term role stability. Respondents emphasized the importance of fellowships, mentoring, and external grant support to build staff capacity. Others noted coordination with university departments as key to navigating hiring and development. Recruitment practices ranged from structured human resources processes to tapping into professional networks and conferences. Respondents also mentioned creating positive work environments, adjusting job roles and titles, and proactively managing staff effort and funding continuity to support team stability. These qualitative findings aligned closely with quantitative findings highlighting the central role of training, mentorship, and workforce development activities across IS centers. Table 5 summarizes the major themes, subthemes, and respondent counts, and verbatim quotes.

#### Question 2: What plans or processes are in place to ensure smooth transitions in leadership?

Across the 13 centers, respondents described varying levels of leadership transition planning and succession readiness. Using inductive thematic analysis, patterns of transition readiness were identified: no formal plan, emerging or informal planning, structured succession (*n* = 4), recently completed transitions (*n* = 2), and absence of planning (*n* = 3).

Several respondents described formal organizational structures to support leadership continuity, including associate director models, transition protocols, leadership pipelines, or operational manuals. Whereas other respondents described centers as relying more heavily on individual leaders or informal mentoring approaches to manage leadership transitions over time.

A few respondents shared recent transitions and the processes used to manage them, while others acknowledged they had no plan in place. These findings suggest variation in organizational maturity and institutionalization across IS centers and programs. Table 6 shows the classifications of succession planning for each of the respondents and representative quotes illustrating how respondents described their center’s current approach.

**Table 6:** Approaches to leadership transitions and succession planning across surveyed IS centers and programs

Patterns	Exemplar Quotes
No formal plan (*n* = 2)	“At this time our Implementation Science Core has one director with no plan for transition in leadership.” (ID 2) “None.” (ID 54)
Emerging or informal planning (*n* = 4)	“We have junior investigators who work closely with the senior leaders. We are giving them opportunities for leadership and preparing them for taking over the program in the future.” (ID 3) “Early stage investigator is in an assistant director role and is actively engaged in core and program initiatives.” (ID 9) “We employ several strategies: 1) Mentoring senior staff to take on more leadership roles in the Center 2) Hiring new senior staff 3) Encouraging professional development opportunities.” (ID 17) “We have hired an early-career faculty investigator with the goal of over time transitioning her into the leadership role.” (ID 30)
Structured planning or succession (*n* = 4)	“We have a leadership structure that includes multiple leaders, and we have a lab manual that documents expectations and responsibilities of all team members, including leadership.” (ID 7) “We have transition plans in place for leaders (e.g., assistant directors, etc.).” (ID 23) “Ensuring inclusion of mid-career faculty in IS Centers leadership structure, ongoing focus on programming for early career individuals to build a pipeline for ongoing expertise.” (ID 37) “We have nested leadership roles… associate directors are strategically chosen to be successors to the director.” (ID 48)
Recent transition completed (*n* = 2)	“We just started talking about ideas for a transition in center director in 2–3 years… We have a ways to go before the plan is set… We executed a plan to replace the long-term administrative coordinator… she mentored the new employee via Zoom (for free) for 4 months after she started.” (ID 10) “Over the past six months, I transitioned to Research Director… and the two Associate Directors became the Center Co-Directors. We also added an Education Director to ensure a strong leadership team.” (ID 46)

*ID 44 was “unsure” of the process

#### Question 3: Any additional insights, challenges, or successes about your program that you believe are important to highlight?

The final open-ended question elicited broader reflections on factors perceived as important to the success and sustainability of their IS centers and programs. Responses were organized into three a priori domains: insights, successes, and challenges, within inductively derived subthemes identified within each category. Table 7 presents the thematic domains, subthemes, respondent counts, and representative quotes.

Insights focused on the value of community and healthcare system partnerships, the evolving visibility of IS within institutions, and the structural importance of CTSA-funding. Respondents described community engagement and collaborative partnerships as foundational to implementation science activities, even though community engagement support was less commonly represented in publicly described service offerings identified through the web-based review. This divergence suggests that some partnership and engagement activities may be operationally important but not consistently reflected in public-facing center descriptions.

Reported successes most commonly related to workforce development, mentorship activities, consultation services, and strategic program growth. Frequently reported challenges included staffing limitations, institutional funding shortfalls, and tensions between academic research timelines and healthcare system operational priorities. Several respondents also described tension between healthcare system operational priorities and the timelines or expectations of academic research. Respondents specifically emphasized the difficulty of sustaining infrastructure and support staff through grant-dependent funding models.

**Table 7:** Insights, successes, and challenges on IS infrastructure from program leaders

Theme	Subtheme	Exemplar Quotes
Insights	Community engagement and partnerships (*n* = 2)	“I believe that strong community partnerships, based on community engagement principles, are critical for our IS activities.” (ID 3) “Project grants are rated highest because they are our primary source of revenue; however, we place an enormous amount of effort and resources toward building community collaborations and other partnerships.” (ID 7)
Insights	Institutional positioning and visibility (*n* = 2)	“As a relatively small core in a health science center, it has taken time to help our institution more broadly understand implementation science and where it can contribute across the translational research spectrum…” (ID 30) “Successes include university-level support and increasing partnerships across schools…” (ID 54)
Insights	CTSA/Infrastructure dependency (*n* = 2)	“The existence of our IS Center is directly tied to our overall CTSA program, without that support, it would likely not exist…” (ID 31) “We have been fortunate to partner closely with our CTSA for many years…” (ID 10)
Successes	Workforce development (*n* = 3)	“Successes - Being able to incrementally build staff capacity in implementation science…” (ID 17) “We have lots of successes with working with more junior faculty in the school…” (ID 29) “Our internal IS team has committed to always supporting K-award applicants with mentorship to grow/maintain an institutional pipeline.” (ID 31)
Successes	Consultation Services (*n* = 2)	“Having methodological cores with expertise in specific aspects of implementation science allow us to do more work including consultations…” (ID 48) “We are a service center that offers consultation services as a shared resource…” (ID 29)
Successes	Programmatic growth and recognition (*n* = 2)	“…launch of a policy implementation science training annual symposium, increased faculty funding…” (ID 54) “…many of those programs were retained in our recent CTSA renewal (7 more years starting summer of 2024)…” (ID 10)
Challenges	Human resources limitations (*n* = 2)	“Hiring staff with experience and expertise in implementation science is challenging…” (ID 17) “We are at a point where none of the core IS faculty can accept more percent effort…” (ID 31)
Challenges	Institutional funding shortfalls (*n* = 2)	“There is always a need for more institutional funding…” (ID 48) “Challenges include sustained funding for support staff.” (ID 54)
Challenges	Health system misalignment (*n* = 1)	“Achieving the buy-in, support, and funding from the health systems for what we do. Often the timing of what they want vs. what we can do in the research is not well aligned.” (ID 23)

## Discussion

This study provides a timely snapshot of IS infrastructure that supports research, training, mentorship, and implementation support within U.S. academic institutions. Drawing from a convergent mixed-methods analysis of 48 centers, including quantitative and qualitative data, our findings reflect a field that has expanded rapidly but now faces critical pressures due to shifting institutional and federal contexts. Policy and administrative changes affecting the stability of federally supported IS initiatives during the 2024–2025 period provide additional context for interpreting the findings. For example, several IS efforts housed within federal agencies, such as those previously supported by the Centers for Disease Control and Prevention (CDC), have experienced restructuring or major funding cuts.

The geographic distribution of identified centers and programs suggests that IS capacity has expanded beyond a small number of institutions and is now represented across all major U.S. regions, although notable regional clustering remains. This pattern may reflect the continued influence of established research-intensive universities and translational science ecosystems in shaping IS capacity nationwide. The concentration of centers in regions with mature academic research ecosystems is also consistent with the broader evolution of IS within translational research infrastructure, including the growing integration of IS into CTSA hubs discussed below.

### Funding dependence, institutionalization, and sustainability

One of the clearest takeaways is the heavy reliance on external federal funding, particularly NIH and CTSA mechanisms, to support operations. Federal funding has historically played a central role and remained the dominant source of support among survey respondents, despite the presence of institutional and healthcare systems partnerships. In light of recent policy changes, reliance on federal funding to support program activities may also pose risks. As of early 2025, the proposed reductions in NIH funding, including indirect cost recovery at 15% (replacing previously negotiated rates of 25 to 70%) has introduced substantial uncertainty for research infrastructure [17].

Although institutional support was commonly reported, grants remained the dominant primary funding source among survey respondents, underscoring the importance of external funding to sustain staffing and operations. These findings suggest that IS centers and programs continue to rely on a combination of external grants and institutional support to sustain core activities. In some cases, infrastructure sustainability appears to depend heavily on external funding and individual leadership instead of stable institutional commitments. Greater institutional investment may be needed to support long-term stability and reduce reliance on external funding.

At the same time, CTSAs may represent more than a funding source. They may be a catalyst for incorporating implementation science into academic medicine. Beginning in approximately 2021, the National Center for Advancing Translational Sciences (NCATS) formally integrated implementation and dissemination science as a core requirement for CTSA hubs. This focus on implementation science within the CTSA Consortium reflects the recognition that IS can help accelerate the movement of evidence from discovery to routine practice. Survey findings from CTSA leaders suggest that implementation science has become an increasingly important component of translational research infrastructure, although the level of integration and support varies across institutions. By connecting translational and implementation scientists earlier in the research pipeline, CTSAs may help strengthen IS capacity, support workforce development, and increase the likelihood that evidence-based innovations achieve broader reach, adoption, and real-world impact. This perspective is consistent with recent calls to embed IS throughout the translational research pipeline rather than treating implementation as a downstream activity that occurs after evidence generation is complete [6, 18, 19].

### Service offerings and funding diversification

IS infrastructure has become embedded within broader translational science and capacity-building missions in academic medicine. The distribution of publicly described service offerings also provides insight into how IS infrastructure is operationalized. Training and consultation services were the most reported offerings, whereas networking activities, pilot funding, and community engagement support were reported less frequently. This pattern suggests that IS centers and programs function primarily as workforce development and methodological support infrastructures rather than as standalone funding entities.

At the same time, findings from both the quantitative and qualitative data suggest opportunities for more diversified infrastructure models. Consultation, training, technical assistance, and implementation support emerge as common functions of IS centers and programs and may provide a foundation for more diversified and sustainable funding models. Potential strategies include fee-for-service consultation models, revenue-generating educational and certificate programs, cost-sharing arrangements with healthcare systems, embedded implementation support within learning health systems, and philanthropic investments focused on workforce development or community engagement.

Similar hybrid approaches have been used already within Clinical and Translational Science Award (CTSA) programs and other academic research cores, where consultation services, pilot funding mechanisms, training activities, and methodological support functions are partially sustained through institutional cost-sharing, service center models, and collaborative partnerships with healthcare systems [4, 6, 7]. Learning health system models and research partnerships may also offer opportunities to align implementation expertise with operational healthcare priorities while supporting longer-term infrastructure sustainability [20, 21].

### Measuring success and structural incentives

Another key finding relates to how centers and programs define and measure success. While respondents selected a range of performance indicators, project-specific grants, publications, funding for mentorship and training opportunities were the most frequently reported measure of success. These priorities were reflected in ranked-choice responses and open-ended comments. Although respondents noted internal collaboration or bibliometric analyses as meaningful metrics, these were exceptions.

The findings raise important questions about how the field defines and incentivizes success and whether those incentives are consistent with real world impact. If implementation science is to fulfill its promise of improving real-world practice, then the ways in which academic institutions evaluate IS programs must evolve. This could include adopting broader metrics that capture practice-based impact, community collaborations, sustainment, and policy influence and implementation outcomes, especially if centers engage more directly with health systems, public health agencies and community partners.

At the same time, the continued reliance on traditional academic metrics likely reflects broader structural realities within academic medicine. Promotion and tenure systems continue to prioritize grants, publications, and scholarly productivity, while funder reporting requirements frequently emphasize measurable short-term outputs. In addition, measuring real-world implementation impact remains methodologically challenging because outcomes such as sustainment, policy influence, and organizational transformation often occur over extended periods and involve multiple interacting systems and stakeholders [22, 23].

### Workforce development and capacity building

Qualitative responses highlighted strategic capacity building through mentorship, consultation, fellowships, career development activities, and workforce training. Workforce development emerged as a prominent theme, with respondents describing efforts to train and mentor early- and mid-career faculty and staff, often through postdoctoral programs, internal seminars, and career development pipelines. These investments in human capital reflect recognition among participating centers and programs that the long-term health of the field depends on developing a skilled and diverse workforce, which is a priority emphasized in recent commentaries on the future of IS training and capacity development [7, 24–26]. They also align with growing recognition of the societal and population health impact of IS, which has increasingly influenced federal funding priorities and training investments. As the field continues to evolve, participating centers and programs described efforts to expand the pipeline of scientists equipped to lead implementation efforts.

Notably, several respondents described mentoring and professional development activities that extended beyond faculty investigators to include staff and operational team members, suggesting a broader and more inclusive conceptualization of implementation science workforce development. These findings align with national efforts to expand D&I training infrastructure through CTSA initiatives, TIDIRH, IS fellowships, and related workforce development programs [4, 7].

### Partnerships, community engagement, and geographic reach

The analysis also highlighted meaningful partnerships with healthcare systems and community organizations. These relationships, frequently reported as key strengths, are central to the applied nature of IS and may also offer untapped opportunities for funding sustainability. Having these real-world ties facilitates translation and provides more resilient platforms for future funding and program alignment. Prior research has shown that strong community and health system partnerships are essential for sustaining implementation activities beyond the life of individual grants [20, 21]. Several respondents also described challenges aligning the timelines and operational priorities of healthcare systems with academic research processes. These tensions may create barriers for sustaining implementation partnerships and responding to rapidly evolving operational needs.

Although relatively few centers publicly described community engagement support on their websites, qualitative responses consistently emphasized community and healthcare system partnerships as central to implementation efforts and organizational success. This discrepancy suggests that some partnership and engagement activities may be operationally important but not consistently reflected in public-facing descriptions of IS infrastructure.

### Leadership continuity and organizational maturity

Lastly, we found wide variation in how centers approach leadership succession and organizational continuity. Some reported formal strategies, including layered leadership structures or associate roles designed to support continuity. Others acknowledged that succession planning was still informal or not yet developed. This is a gap worth addressing, especially as the field becomes more reliant on institutional knowledge and long-term relationships.

As IS infrastructure becomes increasingly reliant on institutional knowledge, collaborative partnerships, and long-term operational relationships, intentional succession planning may be critical to ensuring organizational stability and avoiding disruptions as leaders transition out of their roles [27, 28].

Together, these findings can inform strategic planning efforts by institutions, funders, and academic leaders seeking to strengthen and sustain IS capacity in the face of uncertainty.

## Limitations

Several limitations must be acknowledged. First, our web-based review relied primarily on structured Google searches limited to the top 20 results per search string, and was conducted over a fixed period (November 11–22, 2022). As a result, eligible centers that appeared lower in the search rankings or were established after the fixed dates may not have been included. For example, Duke University’s Implementation Science Research Collaborative (INTERACT) was not identified through the search strategy. Similarly, Duke’s newly awarded CTSA which includes a D&I core for the first time, was launched in August 2025 and was not yet in place during data collection. Although Google searches aligned with our goal of identifying publicly visible IS infrastructure, future studies could strengthen sampling approaches by incorporating NIH RePORTER, PCORI award databases, CTSA hub directories, institutional research core listings, and professional implementation science networks. Nevertheless, the methodological expertise of the professional medical librarian strengthened the rigor of the web-based review through specialized expertise in grey literature searching, search strategy development, and systematic website management procedures, including a structured bookmarking approach adapted from published grey literature search methods [13].

Second, the web-based review and REDCap survey were conducted during different time periods. Because IS infrastructure continues to evolve, organizational structures, funding mechanisms, leadership arrangements, and service offerings may have changed between data collection phases. However, the staggered design allowed survey respondents to verify and update information collected during the earlier web-based review and provide additional context on current operations and sustainability.

Third, the survey response rate (27.1%) was modest with only 13 of 48 identified centers and programs participating. Comparison of respondents and nonrespondents using data from the web-based review suggested broadly similar organizational characteristics, including institution type, focus areas, and external partnerships. Responding centers were more likely to report training, consultation, and community engagement activities; however, these differences likely reflect variation in data completeness rather than true organizational differences. Survey respondents had the opportunity to verify, correct, and supplement information collected during the web review, whereas information for nonresponding centers and programs was derived solely from publicly available websites. Because organizational structures, funding models, partnerships, and service offerings were not independently verified, some differences may reflect variation in the availability and completeness of publicly reported information rather than true organizational differences. Consequently, survey findings should be interpreted as reflecting the experiences and perspectives of participating entities and may not be representative of the broader landscape of academic IS centers and programs in the U.S. Despite these limitations, this study offers one of the most detailed mixed-methods overviews of IS infrastructure to date.

### Future research

Future studies should use more comprehensive approaches to identifying IS centers and programs, including government databases, CTSA hub directories, institutional research core listings, and professional implementation science networks. Expanding eligibility criteria beyond formally designated IS centers and programs may also help capture implementation science activities embedded within academic departments, schools, healthcare systems, public health agencies, and community organizations. Longitudinal research is also needed to examine how IS centers evolve over time in response to changes in funding, institutional priorities, leadership, and workforce needs. Additional work is needed to better understand sustainable models for long-term implementation support across diverse organizational settings.

## Conclusions

This study provides one of the first convergent mixed-methods overview of IS infrastructure in the United States. The findings underscore the central role of training, mentorship, consultation, and workforce development within IS infrastructure, while also identifying ongoing challenges related to funding stability, institutional support, leadership continuity, and the measurement of real-world implementation impact. As the field continues to grow, more sustainable funding approaches, stronger institutional integration, and broader definitions of success may be needed to support long-term IS capacity within academic settings.

## Supplementary Information

Below is the link to the electronic supplementary material.

Supplementary Material 1: Convergent Mixed-Methods Design and Integration Framework.

Supplementary Material 2: Supplementary Table S1. Academic implementation science centers and programs identified through a web-based review (N = 48).

Supplementary Material 3: Supplementary Table S2. Google Search Strings.

Supplementary Material 4: Supplementary Table S3. Data Dictionary and Variable Definitions.

Supplementary Material 5: Supplementary Table S4. Analysis of Survey Respondents and Nonrespondents.

Supplementary Material 6: Supplementary Table S5. Qualitative Analytic Framework: Domains, Themes, and Definitions.

## Data Availability

Data may be available upon request.

## Abbreviations

CDC: Centers for Disease Control and Prevention
CIN: Center of Innovations
CTSA: Clinical and Translational Science Award
CTSI: Clinical and Translational Science Institute
D&I: Dissemination and Implementation
ID: Identifier (used in quotes from survey respondents)
INTERACT: Implementation Science Research Collaborative
IRB: Institutional Review Board
IS: Implementation Science
NIH: National Institutes of Health
PCORI: Patient-Centered Outcomes Research Institute
RQA: Rapid Qualitative Analysis
TIDIRH: Training Institute for Dissemination and Implementation Research in Health
VA: Veterans Affairs
