# Supplementary material for: Implementation science infrastructure in U.S. academic institutions: a convergent mixed-methods study
Source: Implement Sci Commun. 2026 Jul 16;7:206. doi: 10.1186/s43058-026-01046-2 (PMC13689246; doi:10.1186/s43058-026-01046-2)
Supplement: Supplementary file 1 [file 43058_2026_1046_MOESM2_ESM.docx]

**Supplementary Table S1.** Academic implementation science centers and programs identified through a web-based review (n = 48).

| **Institution** | **Center / Program Name** | **State** |
| --- | --- | --- |
| Albert Einstein College of Medicine | Behavioral and Implementation Science Core | NY |
| Albert Einstein College of Medicine | Health Behavior Research & Implementation Science (HBRIS) | NY |
| Boston University | Evans Center for Implementation and Improvement Sciences (CIIS) | MA |
| Brown University | Brown Research on Implementation and Dissemination to Guide Evidence Use (BRIDGE) Program | RI |
| City University of New York (CUNY) | The CUNY Institute for Implementation Science in Population Health (ISPH) | NY |
| Columbia University | Irving Institute for Clinical and Translational Research | NY |
| Columbia University | Implementation Science Lab | NY |
| Cornell University, Weill Cornell Medical College | Weill Cornell Clinical and Translational Science Center | NY |
| Dartmouth College | SYNERGY, Dartmouth's Clinical and Translational Science Institute | NH |
| Emory University | Prevention and Implementation Sciences Core | GA |
| Emory University | Emory Winship Cancer Institute | GA |
| Georgetown/Howard University | The Georgetown-Howard Universities Center for Clinical and Translational Science (GHUCCTS) | DC |
| Harvard University | Implementation Science Center for Cancer Control Equity (ISCCCE) | MA |
| Harvard University | The Center for Healthcare Delivery Sciences (C4HDS) | MA |
| Indiana University | Center for Health Innovation and Implementation Science | IN |
| Indiana University | Indiana Clinical and Translational Sciences Institute | IN |
| Indiana University-Purdue University Indianapolis | Regenstrief Institute | IN |
| Louisiana State University (LSU) | Louisiana Clinical and Translational Science (LA CaTS) | LA |
| Medical College of Wisconsin | Collaborative for Healthcare Delivery Science | WI |
| Medical University of South Carolina | The Dissemination & Implementation Science Collaborative | SC |
| Michigan State University | Flint Center for Health | MI |
| New York University (NYU) | Global Center for Implementation Science | NY |
| Northwestern University | The Northwestern University Clinical and Translational Sciences (NUCATS) Institute | IL |
| Northwestern University | Center for Health Services & Outcomes Research | IL |
| Northwestern/University of Chicago | Behavioral, Social, and Implementation Sciences Core | IL |
| Ohio State University | Fuld National Institute for EBP | OH |
| Old Dominion University | Research and Infrastructure Service Enterprise (RISE) | VA |
| Pennsylvania State University | Penn State Clinical & Translational Science Institute's Implementation Science Core | PA |
| The University of Texas Health Science Center at Houston | UTHealth Institute for Implementation Science | TX |
| University of Arkansas for Medical Sciences | The Center for Implementation Research | AR |
| University of California, Irvine | UCI Institute for Clinical and Translational Science Dissemination and Implementation Unit | CA |
| University of Chicago | NORC Health Implementation Science Center | IL |
| University of Cincinnati College of Medicine | Center for Clinical & Transitional Science & Training | OH |
| University of Colorado | Colorado Clinical and Translational Sciences Institute | CO |
| University of Florida | Division of Implementation Science and Health Interventions in the Department of Health Outcomes & Biomedical Informatics (HOBI) | FL |
| University of Florida | Dissemination and Implementation Science Core | FL |
| University of Illinois at Chicago | Center for Dissemination and Implementation Science | IL |
| University of North Carolina at Chapel Hill | National Implementation Research Network | NC |
| University of North Carolina at Chapel Hill | Cancer Prevention and Control Program | NC |
| University of Pittsburgh | Dissemination and Implementation Science Collaborative | PA |
| University of Texas Health Science Center at San Antonio (UTHSCSA) | Institute for the Integration of Medicine and Science (IIMS)/Center for Research to Advance Community Health (ReACH) | TX |
| University of Texas Health Science Center at San Antonio (UTHSCSA) | Institute for Integration of Medicine and Science (IIMS) | TX |
| University of Washington | The RISE Mental Health Lab | WA |
| University of Washington | The Institute of Translational Health Sciences (ITHS) Dissemination and Implementation (D&I) Program | WA |
| University of Wisconsin | UW Institute for Clinical and Translational Research | WI |
| Vanderbilt University | Center for Clinical Quality and Implementation Research | TN |
| Washington University in St. Louis | Implementation Research Institute | MO |
| Yale University | Yale Center for Methods in Implementation and Prevention Science (CMIPS) | CT |
