# Supplementary material for: Implementation science infrastructure in U.S. academic institutions: a convergent mixed-methods study
Source: Implement Sci Commun. 2026 Jul 16;7:206. doi: 10.1186/s43058-026-01046-2 (PMC13689246; doi:10.1186/s43058-026-01046-2)
Supplement: Supplementary file 2 [file 43058_2026_1046_MOESM3_ESM.docx]

**Supplementary Table S2.** Google Search Strings

Developed by Lesley Skalla, MSLS; Duke University Medical Center Library & Archives, Duke University School of Medicine.

Available online https://medspace.mc.duke.edu/concern/reviews/5x21tg037

**Google search strings**

1. **Implementation science_.edu**

("implementation science" OR "implementation sciences" OR "implementation research") AND (university OR center OR college OR department OR institute OR institution OR collaborative OR program OR core OR hub) site:.edu

2. **Implementation science_.org**

("implementation science" OR "implementation sciences" OR "implementation research") AND (university OR center OR college OR department OR institute OR institution OR collaborative OR program OR core OR hub) site:.org

3. **dissemination science_.edu**

("dissemination science" OR "dissemination sciences" OR "dissemination research") AND (university OR center OR college OR department OR institute OR institution OR collaborative OR program OR core OR hub) site:.edu

4. **dissemination science_.org**

("dissemination science" OR "dissemination sciences" OR "dissemination research") AND (university OR center OR college OR department OR institute OR institution OR collaborative OR program OR core OR hub) site:.org

5. **dissemination and implementation_.edu**

(“dissemination and implementation” OR “dissemination and implementation science” OR “dissemination and implementation sciences” OR “dissemination and implementation research”) AND (university OR center OR college OR department OR institute OR institution OR collaborative OR program OR core OR hub) site:.edu

6. **dissemination and implementation_.org**

(“dissemination and implementation” OR “dissemination and implementation science” OR “dissemination and implementation sciences” OR “dissemination and implementation research”) AND (university OR center OR college OR department OR institute OR institution OR collaborative OR program OR core OR hub) site:.org

7. **outcomes research_.edu**

("outcomes research" OR “outcomes science” OR "outcome research" OR “outcome science”) AND (university OR center OR college OR department OR institute OR institution OR collaborative OR program OR core OR hub) site:.edu

8. **outcomes research_.org**

("outcomes research" OR “outcomes science” OR "outcome research" OR “outcome science”) AND (university OR center OR college OR department OR institute OR institution OR collaborative OR program OR core OR hub) site:.org

9. **delivery science_.edu**

("delivery science" OR “delivery sciences” OR “delivery research”) AND (university OR center OR college OR department OR institute OR institution OR collaborative OR program OR core OR hub) site:.edu

10. **delivery science_.org**

("delivery science" OR “delivery sciences” OR “delivery research”) AND (university OR center OR college OR department OR institute OR institution OR collaborative OR program OR core OR hub) site:.org

11. **translational science_.edu**

("translational science" OR “translational sciences" OR “translational research”) AND (university OR center OR college OR department OR institute OR institution OR collaborative OR program OR core OR hub) site:.edu

12. **translational science_.org**

("translational science" OR “translational sciences" OR “translational research” AND (university OR center OR college OR department OR institute OR institution OR collaborative OR program OR core OR hub) site:.org
