# Supplementary material for: Implementation science infrastructure in U.S. academic institutions: a convergent mixed-methods study
Source: Implement Sci Commun. 2026 Jul 16;7:206. doi: 10.1186/s43058-026-01046-2 (PMC13689246; doi:10.1186/s43058-026-01046-2)
Supplement: Supplementary file 3 [file 43058_2026_1046_MOESM4_ESM.docx]

**Supplementary Table S3**. Data Dictionary and Variable Definitions

Appendix S6 presents the variables used in the study, including data abstracted from institutional websites, survey measures collected through the web-based review and the REDCap survey. Variable definitions and coding schemes are provided to enhance transparency and reproducibility.

| **Original Data Source** | **Variable** | **Description** | **Data Type** | **Code** |
| --- | --- | --- | --- | --- |
| Web-based Review | center_name | Name of center | Text |  |
| Web-based Review | aff_uni | Affiliated with a university | Text |  |
| Web-based Review | assoc_uni | Name of university | Text |  |
| Web-based Review | city | City | Text |  |
| Web-based Review | state | State | Text |  |
| Web-based Review | geo_loc | **Geographic location** | Numeric | 1 ="Midwest", 2 ="Northeast", 3 ="South", 4"West" |
| Web-based Review | pub_pri | **Public or Private** | Numeric | 1=public, 2=private |
| Web-based Review | research_focus | Research focus | Numeric | 0-no, 1-yes |
| Web-based Review | teaching_focus | Teaching focus | Numeric | 0-no, 1-yes |
| Web-based Review | mentorship_focus | Mentorship focus | Numeric | 0-no, 1-yes |
| Web-based Review | p_hcs | Partnered with a health care system | Numeric | 0-no, 1-yes |
| Web-based Review | p_cg | Partnered with a community group | Numeric | 0-no, 1-yes |
| Web-based Review | serv_offer | List Services offered | Text |  |
| Web-based Review | consult | Service Offered: Consultation* | Numeric | 0-no, 1-yes |
| Web-based Review | training | Service Offered: Training* | Numeric | 0-no, 1-yes |
| Web-based Review | tech_assist | Service Offered: Technical assistance (implementation support)* | Numeric | 0-no, 1-yes |
| Web-based Review | pilot_funding | Service Offered: Pilot Funding* | Numeric | 0-no, 1-yes |
| Web-based Review | community | Service Offered: Community Engagment* | Numeric | 0-no, 1-yes |
| Web-based Review | networking | Service Offered: Networking* | Numeric | 0-no, 1-yes |
| REDCap Survey | oeq_pfund_1 | Source1 _Grants | Numeric | 0-no, 1-yes |
| REDCap Survey | oeq_pfund_2 | Source1_Institutional support | Numeric | 0-no, 1-yes |
| REDCap Survey | oeq_pfund_3 | Source1_Government Contracts | Numeric | 0-no, 1-yes |
| REDCap Survey | oeq_pfund_4 | Source1_Tuition or Course Fees | Numeric | 0-no, 1-yes |
| REDCap Survey | oeq_pfund_5 | Source1_Endowments | Numeric | 0-no, 1-yes |
| REDCap Survey | oeq_pfund_6 | Source1_Philanthropic Donations | Numeric | 0-no, 1-yes |
| REDCap Survey | oeq_pfund_7 | Source1_Industry Partnerships | Numeric | 0-no, 1-yes |
| REDCap Survey | oeq_pfund_8 | Source1_Contracts and Consulting Services | Numeric | 0-no, 1-yes |
| REDCap Survey | oeq_pfund_97 | Source1_Other | Text |  |
| REDCap Survey | oeq_ofund_1 | Source2 _Grants | Numeric | 0-no, 1-yes |
| REDCap Survey | oeq_ofund_2 | Source2_Institutional support | Numeric | 0-no, 1-yes |
| REDCap Survey | oeq_ofund_3 | Source2_Government Contracts | Numeric | 0-no, 1-yes |
| REDCap Survey | oeq_ofund_4 | Source2_Tuition or Course Fees | Numeric | 0-no, 1-yes |
| REDCap Survey | oeq_ofund_5 | Source2_Endowments | Numeric | 0-no, 1-yes |
| REDCap Survey | oeq_ofund_6 | Source2_Philanthropic Donations | Numeric | 0-no, 1-yes |
| REDCap Survey | oeq_ofund_7 | Source2_Industry Partnerships | Numeric | 0-no, 1-yes |
| REDCap Survey | oeq_ofund_8 | Source2_Contracts and Consulting Services | Numeric | 0-no, 1-yes |
| REDCap Survey | oeq_ofund_97 | Source2_Other | Text |  |
| REDCap Survey | oeq_perf_1 | Measure1_Center or program grants | Numeric | 0-no, 1-yes |
| REDCap Survey | oeq_perf_2 | Measure1_Project grants | Numeric | 0-no, 1-yes |
| REDCap Survey | oeq_perf_3 | Measure1_Funding mentorship/training opportunities | Numeric | 0-no, 1-yes |
| REDCap Survey | oeq_perf_4 | Measure1_Publications | Numeric | 0-no, 1-yes |
| REDCap Survey | oeq_perf_5 | Measure1_Partnerships | Numeric | 0-no, 1-yes |
| REDCap Survey | oeq_perf_6 | Measure1_Student/faculty engagement | Numeric | 0-no, 1-yes |
| REDCap Survey | oeq_perf_7 | Measure1_Ongoing community collaborations | Numeric | 0-no, 1-yes |
| REDCap Survey | oeq_perf_8 | Measure1_Alumni impact | Numeric | 0-no, 1-yes |
| REDCap Survey | oeq_perf_97 | Measure1_Other | Numeric | 0-no, 1-yes |
| REDCap Survey | oeq_cpg | Measure2_Center or program grants | Numeric | 0-no, 1-yes |
| REDCap Survey | oeq_pg | Measure2_Project grants | Numeric | 0-no, 1-yes |
| REDCap Survey | oeq_fmto | Measure2_Funding mentorship/training opportunities | Numeric | 0-no, 1-yes |
| REDCap Survey | oeq_pubs | Measure2_Publications | Numeric | 0-no, 1-yes |
| REDCap Survey | oeq_part | Measure2_Partnerships | Numeric | 0-no, 1-yes |
| REDCap Survey | oeq_sfe | Measure2_Student/faculty engagement | Numeric | 0-no, 1-yes |
| REDCap Survey | oeq_occ | Measure2_Ongoing community collaborations | Text |  |
| REDCap Survey | oeq_plans | What plans or processes are in place to ensure smooth transitions in leadership? | Text | Open ended - Key Question 1 |
| REDCap Survey | oeq_long | How does your program recruit, retain, and develop staff to support long-term success? | Text | Open ended - Key Question 2 |
| REDCap Survey | oeq_share | Please share any additional insights, challenges, or successes about your program that you believe are important to highlight. | Text | Open ended - Key Question 3 |
