# Supplementary material for: Implementation science infrastructure in U.S. academic institutions: a convergent mixed-methods study
Source: Implement Sci Commun. 2026 Jul 16;7:206. doi: 10.1186/s43058-026-01046-2 (PMC13689246; doi:10.1186/s43058-026-01046-2)
Supplement: Supplementary file 4 [file 43058_2026_1046_MOESM5_ESM.docx]

**Supplementary Table S4.** Analysis of Survey Respondents and Nonrespondents

We compared the 13 survey respondents versus the 35 nonrespondents using information already collected during the web review to answer whether the responding centers (n=13) differed from the nonresponding centers (n=35)?

**Table S4.** Organizational Characteristics of Responding and Nonresponding Centers and Programs

| **Characteristic** | **Respondents (n=13)** | **Nonrespondents (n=35)** |
| --- | --- | --- |
| **Institution Type** |  |  |
| Public | 5 (38.5) | 17 (48.6) |
| Private | 8 (61.5) | 18 (51.4) |
| **Areas of Focus** |  |  |
| Research + Mentorship | 6 (46.2) | 16 (45.7) |
| Research + Mentorship + Teaching | 6 (46.2) | 10 (28.6) |
| Research only | 1 (7.7) | 8 (22.9) |
| Teaching + Mentorship | 0 (0.0) | 1 (2.9) |
| **Partnered with a Healthcare System** |  |  |
| No | 6 (46.2) | 20 (57.1) |
| Yes | 7 (53.8) | 15 (42.9) |
| **Partnered with a Community Group** |  |  |
| No | 6 (46.2) | 20 (57.1) |
| Yes | 7 (53.8) | 15 (42.9) |
| **Service Type** |  |  |
| Training | 10 (76.9) | 16 (45.7) |
| Consultation Services | 9 (69.2) | 13 (37.1) |
| Community Engagement Support | 6 (46.2) | 7 (20.0) |
| Technical Assistance | 5 (38.5) | 7 (20.0) |
| Networking Opportunities | 3 (23.1) | 3 (8.6) |
| Pilot Funding | 2 (15.4) | 1 (2.9) |

Note: Respondent status was defined based on participation in the REDCap survey (n=13). Characteristics of nonresponding centers (n=35) were derived solely from publicly available website information collected during the web-based review. Differences between groups may therefore reflect variation in the completeness of publicly available information rather than true organizational differences.
