# Supplementary material for: Implementation science infrastructure in U.S. academic institutions: a convergent mixed-methods study
Source: Implement Sci Commun. 2026 Jul 16;7:206. doi: 10.1186/s43058-026-01046-2 (PMC13689246; doi:10.1186/s43058-026-01046-2)
Supplement: Supplementary file 5 [file 43058_2026_1046_MOESM6_ESM.docx]

**Supplementary Table S5.** Qualitative Analytic Framework: Domains, Themes, and Definitions

| **Domain** | **Theme** | **Definition** |
| --- | --- | --- |
| Staff Recruitment, Retention, and Development | Institutional Structure for Workforce Development | Recruitment and workforce development activities coordinated through formal organizational units, administrative systems, or implementation science divisions. |
| Staff Recruitment, Retention, and Development | Recruitment Strategies | Approaches used to identify, attract, and hire personnel, including conferences, professional networks, recruitment fairs, and institutional hiring processes. |
| Staff Recruitment, Retention, and Development | Retention Through Job Structure and Stability | Strategies intended to retain personnel through promotion pathways, title advancement, workload management, proactive monitoring of effort allocation, and funding continuity. |
| Staff Recruitment, Retention, and Development | Training Support | Activities that support workforce development through fellowships, mentoring, career development programs, implementation science training opportunities, and other educational initiatives. |
| Staff Recruitment, Retention, and Development | Supportive Organizational Culture | Organizational practices that promote staff engagement, collaboration, participation, leadership opportunities, professional support, and workplace satisfaction. |
| Leadership Transitions and Succession Planning | No Formal Succession Plan | Absence of an identified leadership succession process or transition strategy. |
| Leadership Transitions and Succession Planning | Emerging or Informal Succession Planning | Early-stage succession activities, including mentoring junior faculty, leadership development, and informal discussions regarding future leadership transitions. |
| Leadership Transitions and Succession Planning | Structured Succession Planning | Formal leadership pipelines, documented succession plans, associate director roles, designated successor positions, and intentional leadership development structures. |
| Leadership Transitions and Succession Planning | Recent Leadership Transition | Leadership transitions that had recently occurred or were actively being implemented at the time of data collection. |
| Additional Insights | Community Engagement and Partnerships | Recognition of community partnerships, stakeholder engagement, and external collaborations as important components of implementation science infrastructure. |
| Additional Insights | Institutional Positioning and Visibility | Reflections on organizational visibility, institutional support, recognition, strategic positioning, and perceived value within academic systems. |
| Additional Insights | CTSA and Infrastructure Dependence | Descriptions of reliance on Clinical and Translational Science Award (CTSA) programs or related institutional infrastructure to support implementation science activities, staffing, or operations. |
| Additional Insights | Workforce Development Successes | Expansion of implementation science capacity through mentorship, faculty development, training programs, fellowships, and workforce pipeline-building activities. |
| Additional Insights | Consultation and Implementation Support Services | Provision of consultation, methodological expertise, implementation support, technical assistance, and shared-resource services as indicators of organizational success. |
| Additional Insights | Programmatic Growth and Recognition | Expansion of programs, increased funding, institutional recognition, enhanced visibility, and continued organizational development. |
| Additional Insights | Human Resource Limitations | Difficulties recruiting, retaining, or expanding implementation science personnel and expertise. |
| Additional Insights | Institutional Funding Shortfalls | Insufficient institutional support, staffing resources, infrastructure investments, or long-term funding stability. |
| Additional Insights | Health System Misalignment | Challenges aligning academic implementation science activities with healthcare system priorities, timelines, incentives, and operational needs. |
